# Les cellulites orbitaires: approche diagnostique, thérapeutique et pronostique dans un centre de référence à Tunis, Tunisie (une étude rétrospective sur 109 cas)

**DOI:** 10.11604/pamj.2022.43.64.34807

**Published:** 2022-10-07

**Authors:** Manel Mekni, Jihene Sayadi, Racem Choura, Achraf Fekih, Dhouha Gouider, Aicha Rouatbi, Imene Zghal, Ines Malek, Amel Chebbi, Leila Nacef

**Affiliations:** 1 Service A d´Ophthalmologie, Institut Hedi Raies d´Ophtalmologie de Tunis, Faculté de Médecine de Tunis, Université Tunis El Manar, Tunisie,; 2 Service C d´Ophthalmologie, Institut Hedi Raies d´Ophtalmologie de Tunis, Faculté de Médecine de Tunis, Université Tunis El Manar, Tunisie

**Keywords:** Cellulite orbitaire, classification de Chandler, évolution, pronostic, Orbital cellulitis, Chandler´s classification, evolution, prognosis

## Abstract

La cellulite orbitaire est une pathologie rare. Deux formes anatomo-cliniques sont à distinguer: une forme «bénigne» pré-septale et une forme «grave» rétro-septale. Le but de ce travail était d´analyser le profil épidémiologique, clinique, thérapeutique et pronostique des cellulites orbitaires dans un centre hospitalier de troisième ligne à Tunis, Tunisie. Nous avons mené une étude rétrospective incluant 109 patients hospitalisés pour cellulite orbitaire. Deux groupes ont été définis: le groupe cellulites rétro-septales incluant 42 patients (38,5%) et le groupe cellulites pré-septales comportant 67 patients (61,5%). La moyenne d´âge des patients était de 27,1 ± 34,8 ans. Le sexe ratio H/F était de 0,84 (45,9% des patients de sexe masculin). La sinusite aiguë était la porte d´entrée la plus fréquemment identifiée dans les cellulite rétro-septale (35,7%, n=15) alors que la dacryocystite aiguë était le point de départ le plus fréquent des cellulites pré-septales (23,9%, n=16). Le diabète, l´œil non fonctionnel et la prise préalable d´anti-inflammatoires non stéroïdiens étaient associés à la forme rétro-septale (p=0,007, p=0,022 et p=0,014 respectivement). Une antibiothérapie par voie générale a été instaurée dans tous les cas. Nous avons eu recours à un traitement chirurgical chez 10 malades (23,8%) du groupe cellulite rétro-septale et 5 patients (7,46%) du groupe cellulite pré-septale. Nous avons noté 9 cas de cécité (8,2%), un état de choc septique et un décès. Les facteurs de mauvais pronostic retenus étaient le délai de consultation ≥ 7 jours (ORa = 4,277, IC 95% = 2,504- 32,426, p=0,006) et le stade de Chandler > III (ORa = 7,009, IC 95% = 1,69-51,839, p = 0,029). En conclusion, dans les pays en voie de développement et notamment en Tunisie, les cellulites orbitaires menacent encore le pronostic visuel voire même vital des patients. Une prise en charge précoce à un stade débutant serait le meilleur garant d´une évolution favorable sans séquelles.

## Abstract (en)

Orbital cellulitis is a rare disease. Two anatomo-clinical forms can be distinguished: a preseptal “benign” form and a retroseptal “severe” form. The purpose of this study was to analyze the epidemiological, clinical, therapeutic and prognostic profile of orbital cellulitis in a third-line Hospital in Tunis, Tunisia. We conducted a retrospective study involving 109 patients hospitalized for orbital cellulitis. Two groups were distinguished: the retroseptal cellulitis group including 42 patients (38.5%) and the preseptal cellulitis group including 67 patients (61.5%). The average age of patients was 27.1 ± 34.8 years. The sex ratio M/F was 0.84 (45.9% of male patients). Acute sinusitis was the most frequently identified portal for retroseptal cellulitis entry (35.7%, n=15), while acute dacryocystitis was the most common cause of preseptal cellulitis (23.9%, n=16). Diabetes, non-functioning eye and prior use of non-steroidal anti-inflammatory drugs were associated with retroseptal cellulitis (p=0.007, p=0.022 and p=0.014 respectively). All patients received systemic antibiotic therapy. Ten patients (23.8%) of the retro-septal cellulitis group and 5 patients (7.46%) of the preseptal cellulitis group underwent surgery. Nine cases of blindness (8.2%), a case of septic shock and a case of death were reported. Poor prognostic factors were a time of consultation > 7 days (aOR = 4.277, 95% CI = 2.504-32.426, p = 0.006) and Chandler stage>III (aOR = 7.009, 95% CI = 1.69-51.839, p = 0.029). In developing countries and especially in Tunisia, orbital cellulitis can be sight threatening or even life-threatening. Early management could lead to a favorable outcome without sequelae.

## Introduction

Les infections orbitaires ou cellulites orbitaires sont les pathologies orbitaires primitives les plus fréquentes définies par une inflammation aiguë du tissu cellulo-graisseux orbitaire d´origine infectieuse [1]. Elles sont relativement rares dans la pratique quotidienne de l´ophtalmologiste. Il existe deux formes anatomo-cliniques: la première est la forme superficielle, bénigne ou cellulite pré-septale, en avant du septum orbitaire [2]. La seconde est la forme sévère rétro-septale ou cellulite orbitaire vraie, s´étendant en arrière du septum orbitaire [2,3]. L'incidence de la cellulite orbitaire a nettement diminué ces dernières années dans les pays développés grâce à un meilleur accès à la vaccination, au traitement des infections des voies respiratoires supérieures et à une prise en charge précoce et ciblée de la maladie [4]. Cependant, dans les pays en voie de développement et notamment en Afrique, la cellulite orbitaire représenterait encore une pathologie grave qui menace le pronostic visuel voire vital des patients [5-9]. Le but de notre étude était d´analyser le profil épidémiologique, clinique, évolutif et thérapeutique des cellulites orbitaires dans une population tunisienne.

## Méthodes

**Protocole de l´étude:** il s´agit d´une étude rétrospective portant sur 109 cas de cellulites orbitaires, colligés dans le Service C de l´Institut Hèdi Raies d´Ophtalmologie de Tunis, sur une période de 10 ans entre janvier 2011 et juin 2021. L'étude a adhéré aux principes de la Déclaration d'Helsinki et a été approuvée par le comité d´éthique de l´hôpital Aziza Othmana de Tunis. Un consentement éclairé de tous les participants a été obtenu.

**Population d´étude:** nous avons inclus tous les patients ayant consulté durant la période d´étude pour cellulite orbitaire pré ou rétro-septale. Les patients avec un suivi < 3 mois ainsi que les patients présentant une inflammation orbitaire non infectieuse (tumorale, en rapport avec une maladie de système…) ont été exclus de notre étude.

**Collecte des données:** les données ont été recueillies à partir des dossiers des patients mentionnant l´âge, le sexe, les antécédents d´immunodépression ou de diabète, les antécédents ophtalmologiques, la prise médicamenteuse, l´examen ophtalmologique, l´examen ORL et l´examen général. Nous avons également recueilli les résultats des explorations biologiques et radiologiques, le traitement prescrit, ainsi que l´évolution. Dans notre étude, les patients ont été classés selon la classification de Chandler en deux groupes [10]: le premier groupe présentant une cellulite rétro-septale; le deuxième groupe une cellulite pré-septale.

Les signes cliniques qui ont permis de retenir le diagnostic d´une cellulite rétro-septale étaient: l´exophtalmie, les troubles de l´oculomotricité, l´altération du réflexe photo-moteur afférent ou la baisse de l´acuité visuelle (AV) (non expliquée par une autre pathologie). Les patients chez qui l´exophtalmie était absente ou peu marquée, l´oculomotricité était normale, sans baisse de l´AV, ou d´altération du réflexe photo-moteur afférent, et dont l´évolution à 48 heures était favorable sous traitement, étaient classés comme ayant une cellulite pré-septale. Concernant l´imagerie, la TDM orbito-cérébrale était indiquée devant la présence de signes en faveur d´une atteinte rétro-septale, une évolution défavorable sous traitement ou une impossibilité d´une évaluation clinique en raison d´un œdème occlusif. Elle a permis de préciser le stade anatomo-clinique de Chandler. Nous avons retenu comme critères de mauvais pronostic: Une AV finale < 1/10 et/ou la survenue de complications graves mettant en jeu pronostic vital (état de choc septique; décès).

### Schéma thérapeutique

**Figure 1:**
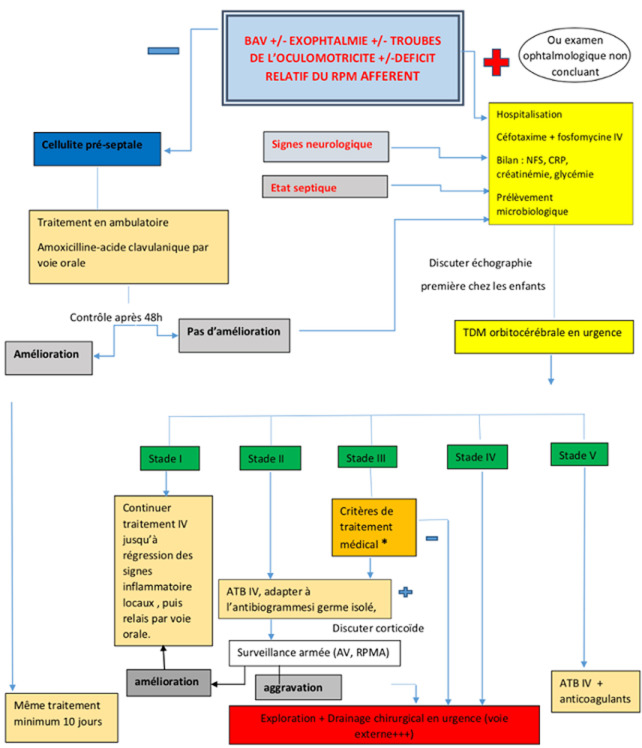
arbre décisionnel devant une inflammation de la région péri-orbitaire

Dans notre centre de 3^e^ ligne, nous avons hospitalisé tous les enfants atteints d´une cellulite orbitaire et tous les adultes du groupe cellulite rétro-septale. Les adultes ne présentant aucun signe d´atteinte rétro-septale étaient traités en ambulatoire avec un contrôle à 48 heures. Tous les patients ont bénéficié d´une antibiothérapie. Les patients du groupe cellulite pré-septale ont reçu le plus souvent en première intention une association amoxicilline-acide clavulanique. Dans le groupe cellulite rétro-septale, le schéma thérapeutique le plus adopté était l´association céfotaxime + fosfomycine par voie intraveineuse. Le métronidazole était associé à chaque fois que des germes anaérobies étaient suspectés. D´autres molécules ont été utilisées moins fréquemment, souvent de 2^e^ intention, tels que les fluoroquinolones, la pépiracilline, l´imipénème, la vancomycine et les aminosides. Le relais par voie orale était assuré classiquement par l´amoxicilline-acide clavulanique, moins fréquemment par la pristinamycine ou l´ofloxacine. Une corticothérapie adjuvante a été prescrite par voie orale, en présence de stigmates d´amélioration, au moins 48h après le début du traitement à la dose de 0,5 mg/kg/j pour une durée de 10 jours. Le traitement chirurgical était de recours en cas de collection, et consistait en une mise à plat d´abcès palpébraux, un drainage de collections orbitaires ou en une éviscération. Notre schéma thérapeutique est résumé dans la Figure 1.

**Analyse statistique:** la saisie des données et leur analyse ont été faites par le logiciel SPSS (version 19.0). L´analyse statistique a permis dans un premier temps de déterminer les caractéristiques des patients. L´analyse univariée a été effectuée à l´aide du test Chi 2 de Pearson ou le test exact de Fischer. Les valeurs quantitatives ont été comparées par le test t de Student. Afin d´identifier les facteurs de mauvais pronostic, nous avons conduit une analyse multivariée en régression logistique méthode pas à pas descendante (à la première étape, on introduit tous les facteurs dont les « p » sont inférieures ou égales à 0,15 et, d´étape en étape, on supprime le facteur qui a le « p » le moins significatif). L´analyse multivariée a permis de calculer des Odds ratios ajustés « ORa », mesurant le rôle propre de chaque facteur. Dans tous les tests statistiques, le seuil de signification a été fixé à 0,05.

## Résultats

### Caractéristiques générales de la population d'étude

Un total de 109 patients a été inclus dans cette étude, répartis en 42 cas de cellulites rétro-septales (38,5%) et 67 cas de cellulites pré-septales (61,5%). La moyenne d´âge des patients était de 27,1 ± 34,8 ans (de 1 à 94 ans). La moyenne d´âge était significativement plus basse dans le groupe cellulite pré-septale: 20,91 ans (de 3 à 83 ans) contre 36,88 ans dans le groupe cellulite rétro-septale (de 1 à 94 ans) (p=0,02). Par ailleurs, 47,4% des patients du groupe cellulite rétro-septale appartenaient à la population pédiatrique contre 58,21% du groupe cellulite pré-septale (p= 0,071). La distribution des cas selon l´âge a montré 2 pics d´incidence dans le groupe cellulite rétro-septale : entre 6 et 16 ans, et après l´âge de 60 ans. Pour les cellulites pré-septale, un seul pic a été constaté aux bas âges (inférieur à 6 ans). L´étude a inclus 50 patients de sexe masculin (45,9%) avec un sexe ratio H/F de 0,84. Le sexe ratio était de 1,1 dans le groupe de cellulite rétro-septale et de 0,76 dans le groupe de cellulite pré-septale.

### Présentation clinique et diagnostic

**Figure 2:**
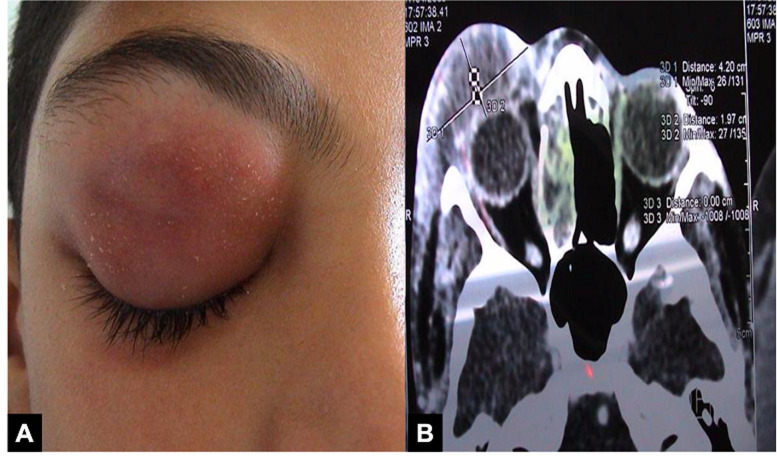
A) patiente âgée de 18 ans qui consulte pour un œdème palpébral droit; B) TDM orbito-cérébrale en coupe transversale montrant une cellulite pré-septale associée à une collection palpébrale (stade I de Chandler)

**Figure 3:**
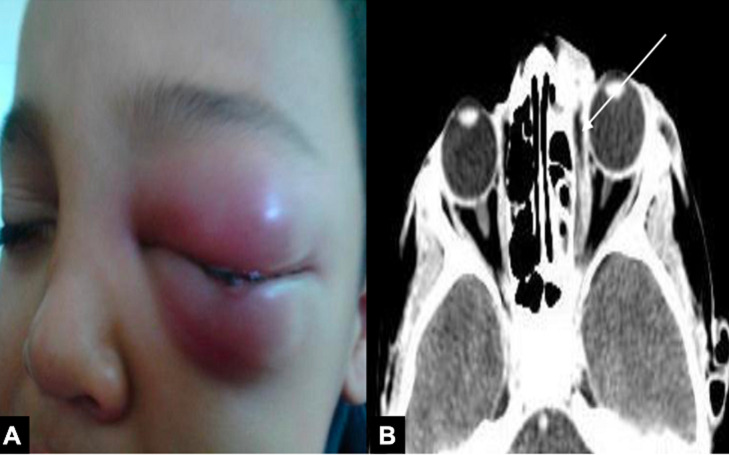
A) patiente âgée de 9 ans qui consulte pour un œdème inflammatoire occlusif de la région orbitaire gauche; B) TDM orbito-cérébrale en coupe transversale mettant en évidence un abcès sous périosté compliquant une ethmoïdite: stade IV de Chandler (flèche blanche)

**Figure 4:**
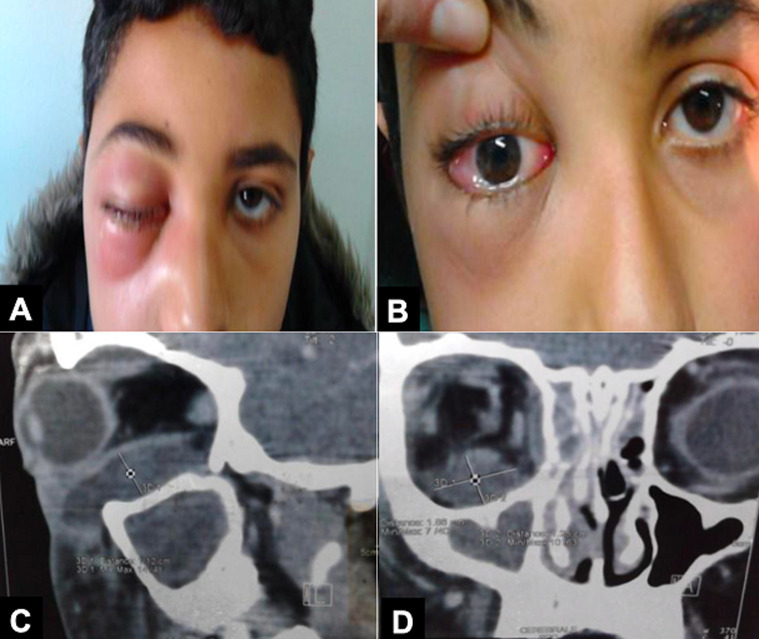
A) patient âgé de 12 ans qui consulte pour une inflammation de la région orbitaire droite; B) l´examen ophtalmologique révélant un chémosis et une ophtalmoplégie droite; C-D) tomodensitométrie (TDM) orbito-cérébrale montrant un abcès orbitaire compliquant une pansinusite (stade IV de Chandler) en coupe sagittale (C) et en coupe coronale (D)

La sinusite aiguë était la porte d´entrée la plus fréquente dans le groupe de cellulite rétro-septale retrouvée dans 15 parmi les 42 cas (35,7%). Le sinus ethmoïdal était le plus impliqué. Les endophtalmies et les panophtalmies étaient des causes assez fréquentes: 5 cas de panophtalmies secondaires à des abcès cornéens (11,9%), 2 endophtalmies endogènes (4,8%) et une endophtalmie post-opératoire (Figure 2). L´atteinte rétro-septale était statistiquement corrélée aux facteurs suivants : le diabète (p=0,07), la prise des anti-inflammatoires non stéroïdiens (p=0,014) et un œil non fonctionnel (p=0,02). Concernant les cellulites pré-septales, la porte d´entrée la plus fréquente était la dacryocystite aiguë (23,9%, n= 16), suivie par les traumatismes palpébraux (16,4%, n=11), les infections cutanées (11,9%, n=8), les piqures d´insectes (10,4%, n=7) et la sinusite aiguë (10,4%, n=7) (Figure 3 et Figure 4). Cliniquement, l´œdème palpébral était un signe constant présent chez tous les patients, suivi par la douleur présente dans 65% des cas (n=71). L´exophtalmie, l´hypertonie et le chémosis étaient significativement associés à la forme rétro-septale (p < 0,001) (Tableau 1). Le taux moyen des globules blancs était comparable entre les deux groupes (p=0,983). Néanmoins, le taux moyen de la CRP était significativement plus élevé dans le groupe cellulite rétro-septale (p=0,019). Le germe le plus fréquemment identifié était le streptocoque (33,3% des germes identifiés). La TDM orbito-cérébrale a été pratiquée chez 66 patients (58,7%). Nous avons analysé les différents signes cliniques du groupe cellulite rétro-septale selon la présence ou non de collection au scanner. Aucun signe clinique n´était statistiquement associé à la présence de collection (Tableau 2).

**Tableau 1:** répartition des signes fonctionnels et des signes cliniques selon le groupe

	Cellulite rétro-septale n (%)	Cellulite pré-septale n (%)	p
**Signes fonctionnels**			
Œdème palpébral	42 (100%)	67 (100%)	-
Douleur	33 (78,6%)	38 (56,3%)	0,02
Baisse de la vision	16 (38,16%)	1 (1,5%)	0
Céphalée	5 (11,9%)	1 (1,5%)	0,032
Diplopie	4 (9,5%)	0 (0%)	0,02
**Signes cliniques**			
BAV	24 (57,14%)	1 (1,49%)	-
Ptose palpébrale	22 (52,38%)	31 (46,26%)	0,53
Exophtalmie	33 (78,57%)	2 (2,98%)	0,00
Trouble de l´oculomotricité	26 (61,9%)	1 (1,49%)	-
Altération du RPM afférent	8 (19%)	0 (0%)	-
Chémosis	23 (57,76%)	9 (13,43%)	0,00
Hypertonie oculaire	17 (40,47%)	2 (2,98%)	0,00

n=nombre de cas; p statistiquement significative si <0,05. BAV: baisse de l´acuité visuelle; RPM: réflexe photomoteur

**Tableau 2:** signes cliniques dans le groupe de cellulite rétro-septale selon la présence ou non de collection au scanner

Signes cliniques	Présence de collection (n)	Absence de collection (n)	P
**Exophtalmie**			0,716
Oui	14	19	
Non	3	6	
**Trouble de l’oculomotricité**			
Oui	10	15	0,680
Non	7	8	
**Ptosis**			0,187
Oui	11	11	
Non	6	14	
**BAV**			
Oui	4	12	0,195
Non	13	13	
**Chémosis**			
Oui	9	14	0,732
Non	8	10	
**HTO**			
Oui	8	9	0,616
Non	9	14	
**Déficit du RPM afférent**			
Oui	2	6	0,439
Non	15	19	

n=nombre de cas; p statistiquement significative si <0,05. BAV: baisse de l´acuité visuelle; HTO: hypertonie oculaire; RPM: réflexe photomoteur

### Prise en charge et suivi

Concernant l´antibiothérapie, l´association céfotaxime-fosfomicyne était la plus adoptée dans le groupe cellulites rétro-septales (35,7%, n=15). Dans le groupe cellulites pré-septales, l´amoxicilline-acide clavulanique était le traitement le plus utilisé (34,3%, n=23). Une corticothérapie par voie orale a été instaurée dans 52,4% des cas (n=22) du groupe cellulite rétro-septale et 22,4% des cas (n=15) du groupe cellulite pré-septale. Deux patients du groupe cellulite rétro-septale ayant une thrombose du sinus caverneux (4,7%) ont bénéficié d´un traitement anticoagulant. Dix patients du groupe cellulites rétro-septales (23,8%) ont nécessité un traitement chirurgical : Un drainage par voie externe a été pratiqué chez 5 patients (11,9%), une éviscération chez 3 patients (7,1%) et une ablation de corps étranger intra-orbitaire dans 2 cas (4,7%). Dans le groupe cellulites pré-septales, 5 patients (7,5%) ont nécessité une mise à plat de collections palpébrales. Le recul variait de 3 mois à 2 ans avec une moyenne de 6 mois. La guérison était la règle pour les cas de cellulite pré-septale. Pour les cellulites rétro-septale, la guérison a été observée dans 73,8% des cas.

### Facteurs pronostiques

Dans notre étude, 11 patients (1%) ont présenté des complications graves: cécité chez 9 patients (8,2%), un état de choc septique et un décès chez une patiente multitarée à 10 jours de son admission par coagulation intravasculaire disséminée (Tableau 3). Les facteurs de mauvais pronostic identifiés à l´analyse univariée étaient: l´âge adulte (p=0,034), un délai de consultation = 7 jours (p=0,02), la présence d´abcès cornéens compliqués (p=0,001) et les stades IV et V de Chandler (p=0,021). A l´étude multivariée, seuls le délai de consultation ≥ 7 jours (ORa = 4,277, IC 95% = 2,504- 32,426, p=0,006) et le stade de Chandler>III (ORa = 7,009, IC 95% = 1,69-51,839, p = 0,029) étaient retenus (Tableau 4).

**Tableau 3:** caractéristiques des patients ayant évolué vers une cécité secondaire à la cellulite orbitaire

	Age	Genre	Antécédents	DDC (jours)	Porte d´entrée	Germes	Stade	Traitement
**1**	94 ans	Féminin	Insuffisance rénale	2	Indéterminée	_	V	Pipéracilline +métronidazole+émipinème +céfotaxime + corticoide+anti-coagulant
**2**	53 ans	Masculin	Diabète	9	Indéterminée	_	IV	Céfotaxime + métronidazole +drainage par voie externe
**3**	78 ans	Masculin	Diabète	30	Sinusite	Mucormycose +Tuberculose	II	Amphotéricine B + INH +rifampicine+ débridement chirugical
**4**	40 jours	Féminin	RCIU, prématurité, Hospitalisée en néonatologie	2	Endophtalmie endogène (Abcès sur veinte)	Pyocyanique	III	Céfotaxime +fosfomycine + métronidazole
**5**	65 ans	Féminin	Diabète	7	Endophtalmie endogène (pyélonéphrite aigue)	Streptocoque	II	Céfotaxime+ fosfomycine+ métronidazole
**6**	60 ans	Masculin	Diabète	8	Abcès cornéen panophtalmie	Streptocoque	IV	Pristinamycine+ amoxi-clav +rifampicine
**7**	41 ans	Masculin	Diabète	45	Abcès cornéen panophtalmie	Staphylocoque+ Fusarium	II	Céfotaxime + ofloxacine +fosfomycine +voriconazole
**8**	80 ans	Masculin	Diabète	6	Endophtalmie post opératoire	-	IV	Céfotaxime+ fosfomycine +métronidazole
**9**	75 ans	Masculin	Diabète	8	Hématogène (Candidose œsophagienne)	Candida	II	Amphotéricine B

DDC: délai de consultation; RCIU: retard de croissance intra-utérin

**Tableau 4:** résultats de l´analyse univariée et de l´analyse multivariée des facteurs pronostiques

	OR non ajusté (IC 95%)	P	OR ajusté (IC 95%)	p
**Âge adulte**	5,523 (1,134-26,894)	**0,034**	-	0,825
**Diabète**	3,750 (0,865-25,971)	0,275	-	-
**Délai de consultation ≥ 7 jours**	5,204 (1,097-24,683)	**0,002**	4,277 (2,504- 32,426)	**0,006**
**Porte d´entrée**				
Abcès cornéen	5,750 (2,341-96,907)	**0,001**	-	0,198
Sinusite	2,645 (0,521-10,375)	0,831	-	-
Infection des paupières	1,567 (0,913-9,174)	0,751	-	-
Piqure d´insecte	1,003 (0,892-5,492)	0,992	-	-
Dacryocystite aigüe	6,353 (0,710-37,113)	0,897	-	-
Traumatisme oculaire	1,102 (0,923-49,235)	0,934	-	-
**Stade anatomo-clinique > III**	6,683 (1,44-45,576)	**0,021**	7,009 (1,69-51,839)	**0,029**

OR: odds ratio; IC: intervalle de confiance; p significative si < 0.05.

## Discussion

Le but de notre travail était d´analyser le profil épidémiologique, clinique, thérapeutique et évolutif des cellulites orbitaires dans une cohorte tunisienne de 109 patients. La cellulite était pré-septale dans 61,5% des cas (67/109) et rétro-septale dans 38,5% des cas (42/109). Le traitement chirurgical était indiqué chez 7,46% des malades du groupe cellulite pré-septale (10/67) et 23,8% des malades du groupe cellulite rétro-septale (5/42). Le pronostic était réservé dans 10,1% des cas (11/109). Sur le plan épidémiologique, en concordance avec Bagheri *et al*. [11], nous avons noté que la moyenne d´âge était inférieure dans le groupe cellulite pré-septale (p=0,02). La distribution de nos patients selon l´âge avait mis en évidence un pic d´incidence aux bas âges pour les cellulites pré-septales et 2 pics pour les cellulites rétro-septales (entre 6 et 16 ans et après 60 ans). Bagheri *et al*. ont noté un seul pic d´incidence dans les deux groupes aux bas âges [11]. Nous expliquons cette différence par la fréquence des comorbidités, notamment le diabète, chez les patients âgés de plus de 60 ans. Contrairement aux pays développés, les endophtalmies et les panophtalmies compliquant les abcès de cornée et les traumatismes oculaires étaient des causes non rares de cellulites rétro-septales dans notre travail (19%). Ceci a été également constaté dans une étude menée en Arabie Saoudite incluant 218 cas de cellulites orbitaires ou les traumatismes étaient identifiés chez 19,7% des patients et les endophtalmies chez 13,3% des cas [12].

Sur le plan clinique, l´exophtalmie, l´hypertonie et le chémosis étaient fortement associés à la forme rétro-septale (p<0,001). Potter *et al*. ont retrouvé des résultats comparables [13]. Nous avons retenu comme facteurs associés à l´atteinte rétro-septale le diabète, l´œil non fonctionnel et la prise préalable des anti-inflammatoires non stéroïdiens (p=0,007, p=0,022 et p=0,014 respectivement). En dehors des atteintes secondaires à la mucormycose, le lien entre cellulites rétro-septales et diabète n´a pas été rapporté dans la littérature [14]. Néanmoins, Colapinto *et al*. ont rapporté un cas de cellulite rétro-septale révélant un diabète et ont recommandé de mesurer systématiquement la glycémie chez les patients présentant ce type d´infection [15]. Dans les cellulites rétro-septales, la TDM est le « gold standard » permettant de différencier une cellulite orbitaire diffuse d´un abcès sous-périosté ou intra-orbitaire dont la prise en charge est différente [16]. Elle a été pratiquée chez 58,7% de nos patients. Le Tableau 5 illustre la distribution des patients selon le stade anatomo-clinique de Chandler dans les différentes études de la littérature.

**Tableau 5:** répartition des cellulites orbitaires selon le stade de Chandler dans les différentes études de la littérature

	Potter_ Australie 2011	Liu_ Taiwan 2006	Botting_New Zeland 2008	Rudloe_Boston 2010	Notre série
**Population**	**Mixte**	**Mixte**	**Pédiatrique**	**Pédiatrique**	**Mixte**
**STADE I**	-	20%	35,18%	42%	37,87%
**STADE II**	64%	48%	37%	17%	34,84%
**STADE III**	22%	0%	22,22%	34%	10,6%
**STADE IV**	14%	32%	3,7%	2%	13,63%
**STADE V**	0%	0%	1,85%	1%	3%

Le traitement de la cellulite orbitaire n´est pas consensuel. Dans notre série, nous avons utilisé l´association amoxicilline-acide clavulanique dans les cellulites pré-septales. Dans des séries pédiatriques, Crosbie *et al*. ont recommandé l´association céfotaxime et fluoxacilline [17], alors que Upile *et al*. ont utilisé l´association Céfotaxime et Métronidazole par voie intraveineuse en première intention dans 92% des cas, avec bonne évolution clinique [18]. Dans les cellulites rétro-septales, l´association céfotaxime et fosfomycine adoptée dans notre série a été recommandée par plusieurs auteurs [18]. Quant à la corticothérapie adjuvante, une méta-analyse récente a conclu qu´elle raccourcissait la durée d´hospitalisation, avec diminution plus rapide de l´inflammation sans risque d´exacerber l´infection [2]. Concernant le traitement chirurgical des stades III et IV de Chandler, les auteurs s´accordent sur la nécessité d´un drainage chirurgical en urgence en cas d´aggravation de l´exophtalmie, de diminution de l´AV ou d´apparition d´une ophtalmoplégie [19]. Dans la littérature, le taux de guérison sans séquelles des cellulites orbitaires varie de 55,8% à 100 % (89,9% des cas dans notre série) [20,21]. Nous avons retenu comme facteurs de mauvais pronostic : un délai de consultation = 7 jours (ORa = 4,277, p=0,006), le stade de Chandler>III, les stades anatomo-cliniques IV et V de Chandler (ORa = 7,009, p = 0,029). D´autres facteurs ont été rapportés tels qu´une AV initiale < 20/200, un déficit du réflexe photomoteur afférent ainsi que l´éloignement géographique et le manque de moyens de transport médicalisé entre les différents services hospitaliers [20,22].

Les caractères rétrospectifs et monocentrique de notre étude constituent ses principales limites. D´autre part, notre échantillon était hétérogène incluant aussi bien des sujets adultes que des enfants. Toutefois, et à notre connaissance, notre étude est la série tunisienne la plus large rapportée à ce jour reflétant les caractéristiques épidémiologiques, diagnostiques, thérapeutiques et pronostiques des cellulites orbitaires.

## Conclusion

En conclusion, dans les pays en voie de développement et notamment en Tunisie, les cellulites orbitaires menacent encore le pronostic visuel et même vital des patients. Une prise en charge précoce à un stade débutant serait le meilleur garant d´une évolution favorable sans séquelles. D´autres études prospectives et multicentriques seraient nécessaires afin de mieux définir les critères pronostiques, stratifier le risque et identifier les cas nécessitant une intervention chirurgicale précoce.

### Etat des connaissances sur le sujet

- Deux formes anatomo-cliniques caractérisent les cellulites orbitaires: la forme pré-septale, généralement bénigne, la forme rétro-septale ou cellulite orbitaire vraie, grave pouvant mettre en jeu le pronostic fonctionnel voire même vital;
- La prise en charge des cellulites orbitaires demeure non encore codifiée;
- Une approche multidisciplinaire est indispensable pour une prise en charge et un suivi optimaux de la maladie.

### Contribution de notre étude à la connaissance

- Le diabète, l´œil non fonctionnel et la prise préalable d´anti-inflammatoires non stéroïdiens seraient associés à la forme rétro-septale de la cellulite orbitaire ;
- Un délai de consultation ≥ 7 jours et un stade de Chandler > I II sont des facteurs de mauvais pronostic ;
- Nous avons proposé dans ce travail un schéma thérapeutique adapté à notre contexte.
